# Health-Related Quality of Life of Asthmatic Patients in Al-Baha City, Saudi Arabia

**DOI:** 10.7759/cureus.53601

**Published:** 2024-02-05

**Authors:** Saleh Jamman M Alzahrani, Haya Abdulaziz K Alzahrani, Shahad Mohamad M Alghamdi, Atheer Nasser A Alzahrani

**Affiliations:** 1 Department of Internal Medicine (Pulmonology and Pulmonary Rehabilitation), King Fahad Hospital, Al-Baha, SAU; 2 College of Medicine, Al-Baha University, Al-Baha, SAU

**Keywords:** asthma, kingdom of saudi arabia (ksa), mini asthma, quality of life, al-baha

## Abstract

Introduction

According to disability-adjusted life years (DALY), bronchial asthma (BA) is rated 28th among the top causes of disease burden globally and among the most significant reasons for years lived with disability. Internationally, 300 million people have asthma, and another 100 million individuals may develop it by 2025. In Al-Baha City, where environmental factors such as dust and pollen levels can exacerbate asthma symptoms, understanding and addressing the health-related quality of life of asthmatic patients is crucial. Understanding the health-related quality of life of asthmatic patients can inform public health policies and initiatives aimed at reducing environmental triggers and promoting better asthma management in the city.

Objectives

The study aims to assess the impact of asthma regarding physical, emotional, and social activities that affect health-related quality of life.

Subjects and methods

A cross-sectional study was conducted from January 2023 to May 2023 at King Fahad Hospital in Al-Baha City, Saudi Arabia. The study used a Mini Asthma Quality of Life Questionnaire that measures physical, emotional, and social activities that affect health-related quality of life.

Results

One hundred and fifty-one out of 185 participants responded, yielding a response rate of 81.6%. The average age of the participants was 52, with a standard deviation of 15.4 years. Participants' responses regarding symptoms related to the environment during the last two weeks indicated "all the time" experiencing feeling bothered by or having to avoid cigarette smoke (n=104, 69%) and dust (n=92, 61%) in the environment. Moreover, considering emotion-related symptoms, 54% reported they did not feel afraid of not having their asthma medication available. Similarly, 46% reported never feeling frustrated because of asthma, whereas 3.3% of the participants documented hardly ever feeling frustrated. Regarding social activity limitations, 44 individuals (29%) reported no limits in these activities, while 43 (28%) reported being completely limited. While there were limitations in work-related activities, 42 participants (28%) reported no restrictions, whereas 34 (23%) reported being completely limited.

Conclusion

The study findings highlight a concern about suboptimal asthma control and the need to attain more satisfactory levels of asthma management.

## Introduction

Bronchial asthma (BA) is a long-term inflammatory process of the airways. Chronic inflammation is attributed to airway hyperresponsiveness, which induces recurring attacks of coughing, wheezing, and dyspnea, especially at night and in the early hours of the morning. These episodes are accompanied by a widespread but diverse airflow blockage in the lungs, which is typically reversible, either spontaneously or with intervention. BA happens at any age, making it one of the most prevalent and significant long-term respiratory illnesses in years, with a worldwide impact [1,2].

According to disability-adjusted life years (DALY), BA is rated 28th among the top causes of disease burden globally and among the most significant reasons for years lived with disability. Internationally, 300 million people have asthma, and another 100 million individuals may develop it by 2025 [3]. The prevalence, severity, and mortality are geographically variable. While the incidence of asthma is higher in high-income nations, low- and middle-income countries account for most asthma-related deaths [4]. Up to the fifth decade of life, the prevalence of asthma remains higher in women than in men [5].

One of the most prevalent chronic illnesses in Saudi Arabia is asthma, and local reports indicate that the condition is becoming more prevalent [6-9]. A previous Saudi study evaluated the impact of asthma on the quality of life of persistent asthmatic patients and its relationship to asthma control and pulmonary function. The results indicated that the total Asthma Quality of Life Questionnaire (AQLQ) was higher in males than females and weakly correlated with age. The Saudi Initiative for Asthma-Asthma Control Test (SINA-ACT) score was significantly correlated with total AQLQ and three domains (activity, symptoms, environmental), but not with the emotional domain. There was no significant correlation between AQLQ and forced expiratory volume (FEV1) or total IgE. Additionally, there was no significant difference in AQLQ and its domains between patients on omalizumab and those not on the treatment, except for the environmental domain, ACT, or FEV1 [7]. A cross-sectional observational study conducted in Saudi Arabia examined the health-related quality of life (HRQoL) of adult asthma and allergic rhinitis (AR) patients in Riyadh, Saudi Arabia. The study compared HRQoL among three groups: asthmatic patients with AR, patients with asthma only, and patients with AR only. Results showed that out of 811 analyzed questionnaires, 23.1% had asthma, 64% had AR, and 27.2% of those with AR also had asthma. The study found a significant association between receiving AR medications and asthma control in respondents with intermittent AR, but no association was observed in respondents with persistent AR. Additionally, the average scores for all eight-item short-form (SF-8) quality of life dimensions were lower in patients with combined asthma and AR compared to patients with AR only and asthma only [8]. Primary care doctors who treat BA patients in Saudi Arabia lack basic knowledge, are unfamiliar with new medications, and fail to recognize the significance of disease control [10,11]. Other factors that affect the severity of a disease burden include socioeconomic status, family size, caregivers' experience, and income [12-15]. Numerous asthma patients have uncontrolled asthma, are still underdiagnosed and inadequately treated, and are at risk of having acute episodes. This causes them to abandon work or school, utilize more expensive intensive healthcare services, and have a lower quality of life [8,16-19]. 

Our searches indicated no research on HRQoL among asthmatics has been conducted in Al-Baha City, but we discovered through monitoring that the Al-Baha region has many asthma cases. Consequently, it is essential to understand the scope and impact of asthma and the implications for HRQoL. 

## Materials and methods

Study design and setting

This study was descriptive and cross-sectional, and it was conducted from January 2023 to May 2023 at King Fahad Hospital pulmonology clinics in Al-Baha City, Saudi Arabia.

Study population

The study population consisted of all male and female asthmatic patients aged 18 years or older who attended our pulmonology clinics from January 2023 to December 2023. Based on their health records, they were clinically stable, with no exacerbations or hospital admissions and no change in the medication in the last four weeks. Potential participants were excluded if they had a chronic respiratory disease other than asthma (e.g., chronic obstructive pulmonary disease (COPD), interstitial lung disease (ILD), bronchiectasis, or tuberculosis (TB)), had mental illnesses or cancer, or did not speak Arabic or English.

Data collection

All patients who met the eligibility criteria were involved in this study after consenting to participate through an electronic questionnaire. One hundred and eighty-five patients were invited to participate in this study. Data was collected using the electronic version of the Mini Asthma Quality of Life Questionnaire (MiniAQLQ). The MiniAQLQ is a disease-specific, 15-item self-administered questionnaire developed and validated in paper and electronic formats. The MiniAQLQ measures functional impairments in four domains: symptoms, activity limitation, emotional function, and environmental stimuli. Participants were asked to recall their experiences over the past two weeks and respond to each question on a 7-point scale (7=no impairment and 1=severe impairment) [20,21]. The questionnaire also gathered demographic information, including age, gender, residence, and associated chronic illness. Due to the lack of a validated Arabic version of MiniAQLQ, the researchers filled out the questionnaire by interviewing the designated individuals through a telephone-based survey.

Ethical approval

The patient's confidentiality and the privacy of their data are the priority. Nothing that leads to ethical issues was used such as names of the participants. The ethical clearance was given by the Scientific Research Committee (Training and Continuing Medical Education Department) of King Fahad Hospital (approval number: KFH\IRB20112022\1). 

Data analysis

The authors submitted the data into a Microsoft Excel spreadsheet, and the data was transferred and analyzed using IBM SPSS Statistics for Windows, Version 26.0 (Released 2019; IBM Corp., Armonk, New York, United States). Testing the association will be done by the chi-squared test. Qualitative variables were represented as percentages and numbers (mean, frequency, etc.) and shown in the figures. A 0.05 level of significance was used in all tests used in the study.

## Results

Demographic characteristics 

A total of 185 individuals were invited to participate in the study, and 151 consented, resulting in a response rate of 81.6%. The average age of the participants was 52, with a standard deviation of 15.4 years. Most of the participants did not smoke (91.4%), were female (76.2%), were Saudi Arabian (96.7%), and resided in areas with moderate altitudes (94.7%). Among the participants, 78 individuals (51.7%) had chronic diseases in addition to asthma; these additional conditions are shown in Table 1. Table 2 provides more detailed information regarding the prevalence of chronic diseases alongside asthma among the study participants.

**Table 1:** Socio-demographic characteristics of the study participants (n=151) IQR: interquartile range

	N (%)
Age, years	
Mean, standard deviation	52±15.4
Median (IQR)	52 (21)
Gender	
Female	115 (76.2%)
Male	36 (23.8%)
Nationality	
Saudi	146 (96.7%)
Non-Saudi	5 (3.3%)
Altitude	
Low-altitude area	8 (5.3%)
Moderate-altitude area	143 (94.7%)
Smoking	
Yes	13 (8.6%)
No	138 (91.4%)
Do you have chronic diseases alongside asthma?	
Yes	78 (51.7%)
No	73 (48.3%)

**Table 2:** Frequencies of chronic diseases alongside asthma among the study participants (n=151)

Specify	
None	73/151 (48.3%)
Allergy	1 (0.6%)
Atrial fibrillation	1 (0.6%)
Benign prostatic hyperplasia	4 (2.6%)
Chronic sinusitis	2 (1.3%)
Diabetes mellitus	33 (21.8%)
Degenerative disc disease	4 (2.6%)
Dyslipidemia	2 (1.3%)
Heart failure	1 (1.3%)
Hypertension	39 (25.8%)
Hypothyroidism	7 (4.6%)
Iron deficiency anemia	1 (0.6%)
Irritable bowel syndrome	1 (0.6%)
Ischemic heart disease	3 (1.9%)
Osteoarthritis	3 (1.9%)
Osteoporosis	2 (1.3%)
Psoriasis	1 (0.6%)
Rheumatoid arthritis	3 (1.9%)
Stroke	1 (0.6%)
Systemic lupus erythematosus	1 (0.6%)
Thalassemia	1 (0.6%)

Participants' responses to the MiniAQLQ 

Table 3 illustrates participants' responses to the MiniAQLQ, specifically regarding symptoms related to the environment. This category, comprising six items, had a mean value of 3.36±1.33. Among these items, the highest mean score of 4.61±1.99 was observed for item Q4, "chest tightness/heaviness feeling," followed by a mean score of 4.25±1.94 for item Q1, "shortness of breath feeling due to asthma." Most participants indicated feeling bothered by or having to avoid cigarette smoke (n=104, 69%) and dust (n=92, 61%) in the environment "all the time."

**Table 3:** Description of HRQoL (MiniAQLQ/environment- and emotion-related symptoms) among the study participants (n=151) MiniAQLQ: Mini Asthma Quality of Life Questionnaire; HRQoL: health-related quality of life; AT: all time; MT: most of the time; GBT: a good bit of the time; ST: some of the time; ALT: a little of the time; HAT: hardly any of the time; NT: none of the time

Items	AT	MT	GBT	ST	ALT	HAT	NT	Mean±SD
Environment-related symptoms								3.36±1.33
Q1: In general, how much of the time during the last two weeks have you felt short of breath as a result of your asthma?	17 (11%)	13 (8.6%)	15 (9.9%)	53 (35%)	13 (8.6%)	4 (2.6%)	36 (24%)	4.25±1.94
Q2: In general, how much time during the last two weeks did you feel bothered by or have to avoid dust in the environment?	92 (61%)	16 (11%)	4 (2.6%)	18 (12%)	6 (4%)	4 (2.6%)	11 (7.3%)	2.25±1.92
Q3: In general, how much of the time during the last two weeks did you feel bothered by coughing?	40 (26%)	21 (14%)	11 (7.3%)	29 (19%)	12 (7.9%)	12 (7.9%)	26 (17%)	3.61±2.2
Q4: In general, how much time during the last two weeks did you experience a feeling of chest tightness or chest heaviness?	11 (7.3%)	15 (9.9%)	18 (12%)	38 (25%)	12 (7.9%)	10 (6.6%)	47 (31%)	4.61±1.99
Q5: In general, how much time during the last two weeks did you feel bothered by/have to avoid cigarette smoke in the environment?	104 (69%)	5 (3.3%)	6 (4%)	11 (7.3%)	6 (4%)	2 (1.3%)	17 (11%)	2.23±2.09
Q6: In general, how much time during the last two weeks did you feel bothered by/have to avoid going outside due to weather or air pollution?	50 (33%)	23 (15%)	18 (12%)	21 (14%)	6 (4%)	10 (6.6%)	23 (15%)	3.21±2.19
Emotion-related symptoms								4.66±1.71
Q7: In general, how much time during the last two weeks did you feel frustrated due to your asthma?	15 (9.9%)	23 (15%)	8 (5.3%)	20 (13%)	11 (7.3%)	5 (3.3%)	69 (46%)	4.85±2.28
Q8: In general, how much time during the last two weeks did you feel afraid of not having your asthma medication available?	18 (12%)	12 (7.9%)	6 (4%)	15 (9.9%)	8 (5.3%)	10 (6.6%)	82 (54%)	5.26±2.26
Q9: In general, how much time during the last two weeks did you have difficulty getting a good night's sleep as a result of your asthma?	28 (19%)	18 (12%)	12 (7.9%)	25 (17%)	9 (6%)	7 (4.6%)	52 (34%)	4.31±2.35
Q10: In general, how much time during the last two weeks did you feel concerned about having asthma?	20 (13%)	11 (7.3%)	11 (7.3%)	21 (14%)	10 (6.6%)	13 (8.6%)	65 (43%)	4.91±2.24
Q11: In general, how much time during the last two weeks did you experience a wheeze in your chest?	20 (13%)	20 (13%)	22 (15%)	40 (26%)	8 (5.3%)	13 (8.6%)	28 (19%)	3.97±1.99

The emotion-related symptoms, comprised of five items, had a mean value of 4.66±1.71. The highest score was observed for item Q8, "experiencing fear about the unavailability of the asthma medication," with a mean of 5.26±2.26. Q10, "feeling concerned about having asthma," had the second highest mean score of 4.91±2.24, followed by Q7, "feeling frustrated due to asthma," with a mean score of 4.85±2.28. Most participants (54%) reported feeling afraid of not having their asthma medication available at some point in the last two weeks. Similarly, 46% reported never feeling frustrated because of asthma, whereas 3.3% of the participants responded that they hardly ever felt frustrated. 

**Figure 1:**
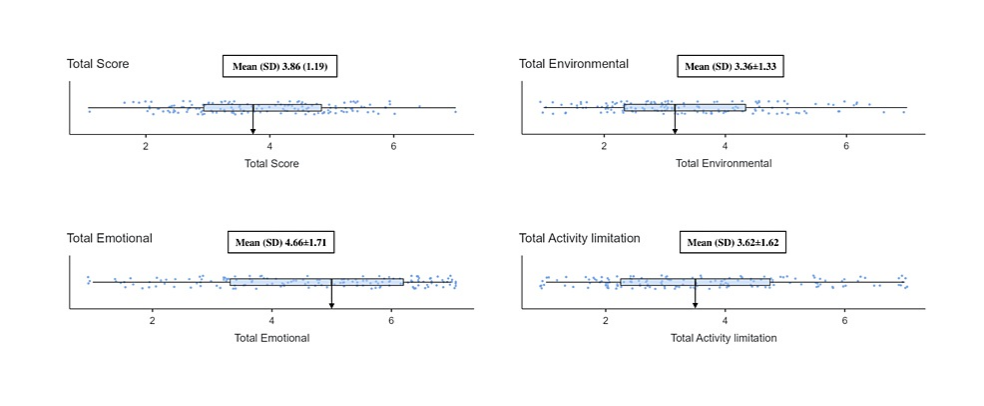
The distribution of the MiniAQLQ total score and its subdomains MiniAQLQ: Mini Asthma Quality of Life Questionnaire

Regarding activity limitations (four items), the average score was 3.62±1.62. The highest score of this subscale was for Q14, with a mean of 3.88±2.44. This item assessed limitations in social activities due to asthma in the last two weeks. Forty-four individuals (29%) reported no limits in these activities, while 43 (28%) reported being completely limited. Q15, which evaluated limitations in work-related activities due to asthma in the last two weeks, had the second-highest mean score at 3.83±2.35. For this item, 42 participants (28%) reported no limits, whereas 34 (23%) reported being completely limited. The overall mean score for the MiniAQLQ was 3.86±1.19, as seen in Figure 1. Additional details regarding activity limitations can be found in Table 4.

**Table 4:** Correlation matrix *: p<0.05; **: p<0.01; ***: p<0.001

		Age	Gender	Altitude	Chronic diseases	Smoking
Total score	Spearman's rho	-0.044	0.271	-0.067	0.036	0.111
	p-value	0.59		0.413	0.658	0.175
	Kendall's tau B	-0.035	0.224	-0.055	0.03	0.092
	p-value	0.535		0.411	0.656	0.174
Total environmental	Spearman's rho	0.004	0.287	-0.112	0.073	0.033
	p-value	0.958		0.171	0.372	0.684
	Kendall's tau B	0.003	0.239	-0.093	0.061	0.028
	p-value	0.961		0.17	0.37	0.683
Total emotional	Spearman's rho	-0.069	0.176	-0.09	0.041	0.11
	p-value	0.404	0.03	0.274	0.615	0.181
	Kendall's tau B	-0.046	0.147	-0.075	0.034	0.091
	p-value	0.416	0.031	0.272	0.613	0.18
Total activity limitation	Spearman's rho	-0.042	0.141	0.06	-0.028	0.109
	p-value	0.611	0.084	0.463	0.737	0.183
	Kendall's tau B	-0.031	0.118	0.05	-0.023	0.091
	p-value	0.586	0.084	0.461	0.736	0.182

The data provided in Table 4 shows that Spearman's rho coefficients for the total score in relation to age, gender, altitude, chronic diseases, and smoking are -0.044, 0.271, -0.067, 0.036, and 0.111, respectively. The corresponding p-values are 0.59, 0.413, 0.658, and 0.175, indicating the significance of these correlations.

## Discussion

Insufficient data on asthma control in the Middle East, particularly in Saudi Arabia, emphasizes the need for comprehensive and uniform data. The current study investigated the HRQoL of 151 individuals with asthma. The study was conducted at King Fahad Hospital in Al-Baha City, Saudi Arabia.

The present findings indicate the considerable influence of asthma on HRQoL; affected participants experienced a moderate reduction in overall HRQoL, as indicated by a mean MiniAQLQ score of 3.86±1.19. Considering the cut-off value for the MiniAQLQ at 4.74, our study revealed that 74.2% of the participants (n=112) exhibited low HRQoL, whereas only 39 individuals demonstrated high HRQoL. This highlights the importance of ongoing patient monitoring and regular evaluation of their quality of life throughout the disease progression.

These results are consistent with previous studies that utilized either the MiniAQLQ or AQLQ tools to evaluate HRQoL among individuals with asthma [22-24]. For instance, using the AQLQ, Oni et al. found that asthmatic patients reported low HRQoL [25]. Similarly, a survey involving over 10,000 patients in the United States revealed poorer HRQoL among asthmatic patients than those without the condition [26]. Nevertheless, higher ratings of HRQoL were documented in several studies conducted in different countries [27-30].

Multiple studies conducted worldwide have documented a positive and independent association between a high level of asthma control and HRQoL [31-33]. Asthma control reflects the impact of the disease on patients, encompassing symptom fluctuations, limitations in daily activities, and the effects of the environment on emotional functioning. Braido et al. provided additional evidence supporting the relationship between asthma control and HRQoL. In their study, approximately one-third of the population attained optimal HRQoL, a change predominantly linked to the asthma control level rather than the duration or severity of asthma [34]. Similarly, Hernandez et al. identified poor asthma control as the sole independent factor affecting HRQoL [35]. The findings indicate that asthma has a considerable influence on HRQoL, with affected participants experiencing a moderate reduction in overall HRQoL.

In Saudi Arabia, a study conducted by BinSaeed [36] showed that only 31.9% of the sample had controlled asthma. Two other studies, one conducted in Saudi Arabia with 1060 participants and another in Jordan with 255 participants, found 36% and 30.6% had well-controlled or completely controlled asthma, as measured by the ACT questionnaire [8,37]. The results from these studies indicate that asthma control plays a paramount role in determining HRQoL among asthmatic patients.

Possible reasons for low asthma control may include failure to implement guidelines effectively, highlighting the need for more practical and consistent guidelines for diagnosis and treatment [38]. By prioritizing effective disease control in patient care, the negative influence of asthma on HRQoL can be mitigated. It is optimal to achieve asthma control, and it is vital to identify and address the key factors that significantly impact populations, allowing patients to lead lives as close to normal as possible.

In the current study, the relationship between gender, the MiniAQLQ score, and the subdomains of the MiniAQLQ exhibited relatively weak correlations. Similarly, factors such as age, altitude, the presence of chronic diseases other than asthma, and smoking were examined for their potential impact on the total MiniAQLQ score and its subdomain scores, except for the activity limitation subdomain. However, no significant correlations were observed between other factors (i.e., age, altitude, presence of chronic diseases other than asthma, and smoking) and the participants' overall MiniAQLQ scores and individual subdomain scores. Nonetheless, future research should consider other potential determinants to comprehensively understand the multifaceted nature of HRQoL in individuals with asthma. The study revealed that a majority of participants exhibited low HRQoL, emphasizing the need for ongoing patient monitoring and regular evaluation of their quality of life throughout the disease progression.

In contrast with the present findings, previous articles identified advanced age as an independent predictor of diminished HRQoL [26,39,40]. Conversely, other studies revealed that younger participants exhibited higher HRQoL scores than their older counterparts [41,42]. Higher education levels were associated with better-controlled asthma in previous research that similarly linked lower education to inadequate control levels [8,36,43]. Smoking is a risk factor for respiratory diseases. However, the present study did not find a significant link between smoking and the total MiniAQLQ score and its subdomain scores, possibly because only 8.6% of our sample were active smokers. In contrast, the majority (n=138, 91.4%) were non-smokers. Turktas et al. found a similar lack of association between smoking and asthma control [44].

Numerous prior studies have documented a noteworthy correlation between the utilization of health insurance and improved HRQoL among asthmatic patients [29,34,35,45-47]. The underlying explanation for this favorable relationship can be attributed to several factors. First, individuals with health insurance tend to exhibit higher levels of adherence to their treatment regimens, potentially due to reduced out-of-pocket medication expenses and increased compliance with treatment guidelines. Second, using health insurance is linked to a lower risk of asthma exacerbations, reducing stress levels and enhancing medication adherence, ultimately leading to minimized hospital costs [48].

Although this study is not the first in Saudi Arabia to investigate the asthma influence on HRQoL among patients, it offers broader insights into its associated factors among the studied population. By recognizing the profound effects of asthma on various aspects of a patient's life, healthcare professionals can develop tailored treatment plans and interventions to improve quality of life outcomes. This finding emphasizes the significance of a patient-centered approach to asthma management, which not only addresses the physiological aspects of the condition but also considers the psychological and social well-being of the patients. Through continuous assessment and proactive measures, healthcare providers can strive to enhance the overall quality of life and well-being of individuals with asthma in the United States.

Strength points and limitations

Our study possesses some strengths that bolster the relevance of its findings. Utilizing a disease-specific tool, the MiniAQLQ, allowed for a more precise and sensitive assessment of asthma's effect on HRQoL. By encompassing multiple dimensions of HRQoL, a comprehensive understanding of asthma's overall effect on HRQoL was achieved. Nonetheless, the present study does have some limitations. The study's reliance on patient recruitment from a single center may restrict the generalizability of its findings. Moreover, the convenience sampling technique may have introduced selection bias, and this cross-sectional study design cannot establish cause-effect relationships.

## Conclusions

The study findings highlight a prevailing concern about suboptimal asthma control within the studied population. This finding emphasizes the pressing need for concerted efforts from healthcare professionals and patients alike to attain more satisfactory levels of asthma management. Addressing the challenges associated with asthma control requires a multifaceted approach that involves enhanced patient education, regular monitoring of symptoms, and adherence to prescribed treatment regimens. Healthcare providers are crucial in fostering patient empowerment through personalized asthma action plans and continuous support. Encouraging self-management practices and proactive communication between patients and their healthcare teams can promote a collaborative and practical approach to asthma control. Moreover, public health initiatives to raise awareness about asthma and its management could improve overall asthma outcomes across the Saudi population. By recognizing the significance of these efforts, the healthcare community can strive to elevate the standards of asthma care and enhance the quality of life for individuals living with this chronic condition in Saudi Arabia. 
